# Editorial for the Special Issue on Advances in Optofluidics

**DOI:** 10.3390/mi9060302

**Published:** 2018-06-15

**Authors:** Xuming Zhang

**Affiliations:** Department of Applied Physics, The Hong Kong Polytechnic University, Hong Kong 999077, China; apzhang@polyu.edu.hk

Optofluidics grew up from the early attempts to integrate optics with microfluidics to exert the benefits of both. Over the last decade, it has made steady progress from the passive interaction of light and liquid media such as liquid waveguides, optical sensors and liquid tunable lenses to the active interaction of photons and liquids such as lasers, particle manipulations and photoreactions. This special issue of Micromachines, entitled “*Advances in Optofluidics*”, collects 9 review articles that update the latest progress of the optofluidics in a broad range of topics, such as droplets, light manipulation, display, refractometry, microcavities and tunable lenses.

One of the core parts of optofluidics is to use liquids as the optical media. Two review articles cover this topic. Yang et al. discussed how to use inhomogeneous liquids to form refractive interfaces for light focusing, or to generate gradient refractive index profiles for advanced effects such as self-imaging, discrete diffraction and optical cloaking [1]. In the other article, Chen et al. narrowed down to a more specific topic—tunable liquid lenses for in-plane light focusing/diverging [2]. They categorized the lenses based on the operation mechanisms and presented their applications in integrated lab-on-a-chip systems for particle trapping and flow cytometry.

Another core part of optofluidics is to use light to measure the change in the liquid media. This special issue has two review articles in this topic. Jian et al. reviewed the recent work on optofluidic refractometry and elaborated different sensing mechanisms/structures and the performance enhancement [3]. In addition, Wang et al. focused on the use of optofluidics to monitor the water quality such as heavy metal, organics, and microbial pollution [4]. This is a new but important area and is worth more exploration. 

Droplet microfluidics aims to discretely manipulate tiny volume of fluids. The use of light enables the droplet sensing and manipulation. In this special issue, El Abed reviewed recent developed methods for real-time analysis of droplet size and size distribution, for active merging of microdroplets using light, or for optical probing [5]. Huang et al. reviewed another aspect—passive micromixing in droplets [6], covering the element designs, the analysis methods and the numerical models. 

Optofluidic microcavities confine both liquid and light in a tiny space and significantly enhance their interaction, especially the active interaction of photons and liquid media. Along this line, Feng et al. summarized the recent advances in the liquid microlasers and their biochemical sensing applications [7]. This review article categorized the laser structures of optical resonant cavities and classified the active and passive sensors.

Optofluidics is often based on a microchip. In fact, it can also utilize other supporting structures with air channels, for instance, microstructured optical fibers. Shao et al. reviewed the recent progress in this interesting and useful topic and discussed various sensing applications [8].

Optofluidic display has become a hot topic in recently years thanks to the brilliant idea and the bright market prospect. As an expert of this field, Shui et al. elaborated the working principles and device structures of three types of reflective displays, and summarized the optofluidic behavior and the controlling factors [9]. Display is one of the areas that bear the hope of real, impactful application of optofluidics. This review article lays down the technical bases and serve as the guide for other researchers.

Certainly, there are some overlaps among these review articles. For instance, the review of optical sensors may partially cover the optical structures, and the application discussions of microcavities and droplets have to involve optical sensors. In view of the completeness of each individual review article, such an overlap is inevitable. Fortunately, the overlap is minor because each article has its own focal interests, which are different from those of the other articles.

I would like to thank all the authors for their great contributions to this special issue. Sincere appreciation also goes to all the reviewers for their efforts and visions to ensure the quality of review articles.

## References

[1] Zuo Y., Zhu X., Shi Y., Liang L., Yang Y. (2018). Light Manipulation in Inhomogeneous Liquid Flow and Its Application in Biochemical Sensing. Micromachines.

[2] Chen Q., Li T., Li Z., Long J., Zhang X. (2018). Optofluidic Tunable Lenses for In-Plane Light Manipulation. Micromachines.

[3] Li C., Bai G., Zhang Y., Zhang M., Jian A. (2018). Optofluidics Refractometers. Micromachines.

[4] Wang N., Dai T., Lei L. (2018). Optofluidic Technology for Water Quality Monitoring. Micromachines.

[5] Hayat Z., El Abed A.I. (2018). High-Throughput Optofluidic Acquisition of Microdroplets in Microfluidic Systems. Micromachines.

[6] Chen C., Zhao Y., Wang J., Zhu P., Tian Y., Xu M., Wang L., Huang X. (2018). Passive Mixing inside Microdroplets. Micromachines.

[7] Feng Z., Bai L. (2018). Advances of Optofluidic Microcavities for Microlasers and Biosensors. Micromachines.

[8] Shao L., Liu Z., Hu J., Gunawardena D., Tam H.-Y. (2018). Optofluidics in Microstructured Optical Fibers. Micromachines.

[9] Jin M., Shen S., Yi Z., Zhou G., Shui L. (2018). Optofluid-Based Reflective Displays. Micromachines.

